# Berry flavonoids are differently modulated by timing and intensities of water deficit in *Vitis vinifera* L. cv. Sangiovese

**DOI:** 10.3389/fpls.2022.1040899

**Published:** 2022-10-31

**Authors:** Giacomo Palai, Giovanni Caruso, Riccardo Gucci, Claudio D’Onofrio

**Affiliations:** Department of Agriculture Food and Environment, University of Pisa, Pisa, Italy

**Keywords:** anthocyanins, deficit irrigation, flavonols, grapevine, phenylpropanoids, stilbenes, transcription factors, transcriptomics

## Abstract

In this work, we tested the effect of different regulated deficit irrigation (RDI) regimes on berry flavonoid content and its relative biosynthetic pathways. Vines were subjected to six irrigation regimes over two consecutive years: a) full irrigation during the entire irrigation period (FI); b) moderate (RDI-1M) or c) severe (RDI-1S) water deficit between berry pea-size and veraison; d) severe water deficit during the lag-phase (RDI-LS); and e) moderate (RDI-2M) or f) severe (RDI-2S) water deficit from veraison through harvest. Berries from both RDI-1 treatments showed the highest accumulation of anthocyanins, upregulating the expression of many genes of the flavonoid pathway since the beginning of veraison until harvest, far after the water deficit was released. Although to a lesser degree than RDI-1, both post-veraison water deficit treatments increased anthocyanin concentration, particularly those of the tri-substituted forms, overexpressing the *F3′5′H* hydroxylases. The moderate deficit irrigation treatments enhanced anthocyanin accumulation with respect to the severe ones regardless of the period when they were applied (pre- or post-veraison). The water deficit imposed during the lag-phase downregulated many genes throughout the flavonoid pathway, showing a slight reduction in anthocyanin accumulation. The measurements of cluster temperature and light exposure highlighted that under deficit irrigation conditions, the effects induced by water stress prevailed over that of light and temperature in regulating anthocyanin biosynthesis. Flavonol concentration was higher in RDI-1S berries due to the upregulation of the flavonol synthases and the flavonol-3-*O*-glycosyltransferases. In this case, the higher cluster light exposure induced by water deficit in RDI-1S berries had a major role in flavonol accumulation. We conclude that the timing and intensity of water stress strongly regulate the berry flavonoid accumulation and that proper management of deficit irrigation can modulate the phenylpropanoid and flavonoid pathways.

## Introduction

Wine grapes are often grown in regions characterized by dry and warm summers where irrigation is needed to support vine growth and yield. A proper irrigation strategy is essential for obtaining high-quality berries and wines (Van Leeuwen et al., 2009; Buesa et al., 2021; Romero et al., 2022; Palai et al., 2022). The primary metabolism of grape berry is strongly affected by deficit irrigation strategies. For instance, sugars and organic acids are positively and negatively correlated, respectively, with the water stress experienced by vines (Mirás-Avalos and Intrigliolo, 2017; Gambetta et al., 2020). Water deficit has been also used to modulate the concentration of secondary metabolites that confer important sensory features to grapes and wines (Bouzas-Cid et al., 2018; Vilanova et al., 2019; Romero et al., 2022). In particular, the triggering effect on the biosynthesis of phenylpropanoids, such as anthocyanins, flavonols, and proanthocyanidins, is one of the major consequences induced by deficit irrigation (Gambetta et al., 2020). A more intense pigmentation in red wines has been previously linked to a higher berry anthocyanin concentration induced by water deficit (Matthews and Anderson, 1989; Deluc et al., 2009; Koundouras et al., 2009; Intrigliolo et al., 2012). Previous studies carried out on different cultivars reported higher concentrations of anthocyanins in berries from grapevines subjected to early water deficit with respect to those sampled from fully irrigated ones (Castellarin et al., 2007; Palliotti et al., 2014; Shellie, 2014; Caruso et al., 2022). Similarly, an increment in berry anthocyanin accumulation was also observed in vines subjected to post-veraison water deficit (Girona et al., 2009; Ollè et al., 2011; Villangò et al., 2016). Nevertheless, since other studies did not show any significant modification in anthocyanin concentration under different water deficit regimes (Petrie et al., 2004; Intrigliolo et al., 2016; Herrera et al., 2017; Brillante et al., 2018), this response, albeit common, is not univocal. The climatic conditions and the genotype, as well as the magnitude and timing of the water deficit imposed, determine the effects on berry anthocyanins by modifying their biosynthetic pathway (Koundouras et al., 2009; Pinasseau et al., 2017; Buesa et al., 2019). Moreover, although most evidence did not indicate a clear link between water stress and flavonol accumulation (Martinez-Luscher et al., 2014; Yu et al., 2016; Savoi et al., 2017), higher flavonol concentrations under deficit irrigation have also been reported (Ojeda et al., 2002; Deluc et al., 2009; Savoi et al., 2016; Torres et al., 2022).

Despite many studies investigated the effect of water deficit on berry flavonoids, only a few have focused on the transcriptomic modulation of the related biosynthetic pathways. Castellarin et al. (2007) reported a greater and earlier expression of the *F3H*, *DFR*, *UFGT*, and *GST* genes of the flavonoid pathway in Cabernet Sauvignon, especially in berries from vines subjected to early moderate to severe water stress. Moreover, they reported a significant upregulation of flavonoid-3′-5′-hydroxylase (*F3′5′H*) genes, as confirmed by Yang et al. (2020) on the same cultivar in berries from deficit (30% ET_c_) irrigated vines from the beginning of the vegetative season to 30 days before harvest. In Merlot, a sustained deficit irrigation promoted the expression of the branch of the flavonoid pathway which leads to the production of tri-hydroxylated anthocyanins (Savoi et al., 2017). Three deficit irrigation treatments at different ET_c_ thresholds (60%, 70%, and 80% ET_c_) upregulated many genes of the flavonoid pathway, of which *PALs*, flavonoid-3′-hydroxylases (*F3′Hs*), and *F3′5′Hs* were closely correlated with anthocyanin accumulation (Ju et al., 2019). It is well known that many of these genes modulated in the flavonoid pathway by water availability are strongly regulated by abscisic acid (ABA) (Pilati et al., 2017; Guo et al., 2021). Transient increases in the expression of the flavonol synthase (*FLS*) genes have also been reported under water deficit (Ojeda et al., 2002; Deluc et al., 2009). In white grapes, the overexpression of an *FLS* was observed after veraison in berries from sustained deficit irrigated vines (Savoi et al., 2016). Nevertheless, the regulation of *FLS* was particularly regulated by light, especially through the transcription factor (TF) *MYBF1* which in turn is controlled through the signaling cascade derived from the photoreception of UV-B radiation (Czemmel et al., 2009; Matus, 2016).

We hypothesize a different biosynthetic modulation of berry flavonoid accumulation induced by the water deficit applied at different phenological stages. To test this hypothesis, we subjected grapevines (cv. Sangiovese) to five regulated deficit irrigation (RDI) treatments and compared them with fully irrigated vines during two consecutive growing seasons. In particular, we imposed a moderate and a severe water deficit either before or after veraison, and we also tested an RDI treatment consisting of a severe water deficit applied only during the lag-phase. The main objective of this study was to evaluate the effect of these RDI strategies on berry flavonoid content and on the modulation of the relative biosynthetic pathways.

## Materials and methods

### Plant material and experimental conditions

The study was carried out over two consecutive years (2019–2020) on 7-year-old grapevines (*Vitis vinifera* L. cv. Sangiovese) grafted on 110 Richter (*Vitis berlandieri* × *Vitis rupestris*) at the experimental farm of the Department of Agriculture Food and Environment of the University of Pisa (43.732153N; 10.465836E). Vines were grown under open-field conditions in 50-L containers (40% peat and 60% silty-loam soil), aligned in north–south-oriented rows and spaced at 4.2 m × 0.9 m distances. All vines were trained according to the Guyot system and were winter pruned leaving one spur with two count buds and one cane with five to seven count buds. Vines were grown inside a trellis system with a cane training wire positioned at 0.9 m from the soil, and shoots were vertically positioned within three pairs of catch galvanized steel. The containers were covered with plastic film from the beginning of the irrigation differentiation in order to minimize soil water evaporation and to exclude rainfall. Each vine was fertilized *via* fertigation three times (two applications between bud break and the berry pea-size stages and one application 1 week after harvest) with about 50 g of NPK 20-20-20 + microelements. Phenological stages were monitored using a modified Eichhorn–Lorenz (EL) scale (Coombe, 1995) (**Table 1**). Harvest dates were established based on the berries’ total soluble solids (TSS) threshold (22 ± 0.5 °Brix) in order to avoid possible influences of sugar content on berry glycosylated compounds. Climatic conditions were monitored using a WatchDog (Spectrum Technologies Inc., Aurora, IL, USA) weather station located on-site.

**Table 1:** Dates of phenological stages of Sangiovese grapevines (*Vitis vinifera* L.) and irrigation volumes applied in 2019 and 2020.

Year	Irrigation treatment	Pea-size (DOY)	Veraison (DOY)	Harvest (DOY)	Irrigation volume (L vine^−1^)			
					PS-L	L-V	V-H	PS-H
2019	FI	163	211	267	166	45	225	436
	RDI-1S	163	217	262	52 (31)	14 (31)	181 (80)	247 (57)
	RDI-1M	163	217	258	91 (55)	27 (60)	165 (73)	283 (65)
	RDI-LS	163	211	266	166	17 (38)	222 (99)	405 (93)
	RDI-2S	163	211	269	166	45	69 (31)	280 (64)
	RDI-2M	163	211	253	166	45	109 (48)	320 (73)
2020	FI	156	208	259	181	48	214	443
	RDI-1S	156	217	259	55 (30)	16 (33)	179 (84)	250 (56)
	RDI-1M	156	212	254	94 (52)	29 (60)	181 (85)	304 (69)
	RDI-LS	156	205	261	181	18 (38)	235 (110)	434 (98)
	RDI-2S	156	208	264	181	48	81 (38)	310 (70)
	RDI-2M	156	208	257	181	48	122 (57)	351 (79)

The percentage of irrigation volume applied to RDI vines with respect to fully irrigated ones (FI) is reported in brackets.

DOY, day of the year; PS-L, pea-size—lag-phase; L-V, lag-phase—veraison; V-H, veraison—harvest; PS-H, pea-size—harvest.

### Irrigation and vine water status

Vines were irrigated three times every day using drip lines (two emitters for each container, 2 L/h each). All vines were fully irrigated until berries reached the pea-size stage (EL 31) [day of the year (DOY) 163 and 156 in 2019 and 2020, respectively], when six irrigation regimes were imposed (**Table 1**). Control vines (FI) were irrigated based on the actual water consumption of the vines, measured as daily weight loss of four representative vines (Caruso et al., 2022) and adjusted in order to maintain the stem water potential (*Ψ*_stem_) above −0.5 MPa. Severe (S) or moderate (M) RDI regimes were applied from berry pea-size until the beginning of veraison (EL 35) (RDI-1S, 30%–31% of FI; RDI-1M, 54%–56% of FI, respectively) and from the beginning of veraison until the harvest (RDI-2S, 31%–38% of FI; RDI-2M, 48%–57% of FI, respectively). A severe RDI treatment was imposed during the lag-phase (RDI-LS, 38% of FI). All RDI vines were fully irrigated when water deficit was not imposed and from harvest until leaf fall (**Table 1**). Irrigation volume applied to FI vines from berry pea-size to harvest was similar in both years (**Table 1**). Similar irrigation volumes were applied in both years according to the RDI treatments (247, 283, 280, 320, and 405 L per vine in RDI-1S, RDI-1M, RDI-2S, RDI-2M, and RDI-LS, respectively, in 2019 and 250, 304, 310, 351, and 434 L per vine in 2020) (**Table 1**). Vine water status was determined on five vines per treatment measuring *Ψ*_stem_ during the entire irrigation period (13 times in both years) at 7–10-day intervals using a Scholander pressure chamber (PMS Instruments, Albany, OR, USA) as reported in Palai et al. (2021). The *Ψ*_stem_ was measured on one fully expanded leaf per vine inserted near the trunk of the vine and covered with aluminum foil for at least 1 h before measurements to block leaf transpiration.

### Canopy growth, cluster temperature, yield, and berry characteristics

The leaf area index (LAI) was estimated 6 and 9 times during the growing season in 2019 and 2020, respectively, on nine vines per irrigation treatment using a canopy analysis system (SunScan model SS1-R3-BF3, Delta-T Devices, Cambridge, UK). PAR was measured at ground level at 0.2-m intervals starting from the center of the row below the canopy through the middle of the interrow using a linear probe positioned parallel to the trellis. The total leaf area per vine (TLA) was estimated from LAI measurements and considering the surface area allocated for each vine. The pruning weight of 15 vines per treatment was determined at the end of February 2020 and 2021 and was used to calculate the yield-to-pruning weight ratio for each vine.

Cluster temperature was continuously monitored in 2020 using a thermocouple placed inside the cluster (three thermocouples per irrigation treatment) since the fruit set. Data were recorded by a data logger (GMR instrument, Florence, Italy) at 5-min intervals. In addition, the diurnal patterns of incident radiation and cluster temperature were simultaneously measured at three different dates in 2020 (green phase, DOY 174; lag-phase, DOY 196; ripening phase, DOY 234). The measurements were carried out at six different times during daylight (08:00, 10:00, 12:00, 14:00, 16:00, and 18:00) on 25 randomly selected clusters per treatment. Cluster temperature was measured with a handheld infrared thermometer (Fluke 568, Everett, WA, USA) on the light-exposed cluster side. Incident radiation was measured using a MultispeQ (Photosinq Inc., East Lansing, MI, USA) as an average of five measurements per cluster taken on the top and on the four exposed sides of the cluster, approximating it to a parallelepiped. Moreover, in order to consider the potential effect of berry size on cluster compactness, and in turn on its temperature, at the same dates, the berry diameter was measured with a digital caliper.

Clusters were harvested from each of the 15 monitored vines per treatment and immediately weighted for yield determination. The TSS, titratable acidity (TA), and pH were determined on 15 vines as well as berry fresh weight (FW) which was measured within 1 h from harvest (one sample of 30 berries from five consecutive vines, for a total of three samples per irrigation treatment). The berry juice was extracted from each sample, TSS was measured with a digital refractometer (DBRwine, HM Digital Ltd., Seoul, Korea), pH was measured with a pH meter (Hanna Instruments, Woonsocket, RI, USA), and a 10-ml aliquot was titrated with 0.1 N of NaOH to an endpoint pH of 8.2 to determine TA.

### Flavonoid determination

Skin anthocyanins and flavonols were determined at DOY 216, 227, 239, and 252 and DOY 213, 221, 230, and 245 in 2019 and 2020, respectively, on three replicates of 30 berries per irrigation treatment immediately frozen in liquid nitrogen after sampling in the field. We added a last date of measurement coinciding with the harvest date, according to **Table 1**. Skin samples were separated in the laboratory just before the analyses. The concentrations of anthocyanins and flavonols were expressed as µg/g of skin FW, equivalents of malvidin-3-glucoside and quercetin-3-O-glucoside, respectively. They were determined by means of HPLC analysis and following a slightly modified procedure reported by Downey and Rochfort (2008) using an Agilent HPLC1260 system with DAD detection and a Poroshell 120 EC-C18 column (4.6 mm × 150 mm, 2.7 μm) (both from Agilent Technologies, Palo Alto, CA, USA). In brief, about 0.4 g of frozen ground grape skin powder was extracted at room temperature with 2 ml of 30% methanol in water. After centrifugation, the supernatant was filtered with a nylon filter (0.22 µm) and injected into the HPLC system. Chromatographic separation was achieved by using a linear gradient from 10% formic acid in water to 10% formic acid in methanol, at a flow rate of 0.8 ml/min. Anthocyanins and flavonols were monitored by DAD detection at 520 and 354 nm, respectively. Identification of the individual component peaks was performed by making a comparison of retention times and UV–VIS absorption data with those found in the literature (Downey and Rochfort, 2008). Quantification was performed by using malvidin-3-glucoside and quercetin-3-glucoside as an external standard to build the calibration curve for anthocyanins and flavonols, respectively.

### RNA extraction and RNA-seq analysis

Transcriptome analysis was carried out in 2020 on berries from FI, RDI-1S, RDI-LS, and RDI-2S treatments. Berry sampling was carried out at the beginning of veraison (except for RDI-2S), at harvest (according to **Table 1**), and at an intermediate date (DOY 234, mid ripening). Three 50-berry replicates per irrigation treatment were randomly collected and immediately frozen in liquid nitrogen. Pedicels and seeds were carefully removed, and the rest of the berries (pulp and skin) were ground in liquid nitrogen. Total RNA was extracted with the Spectrum Plant total RNA kit (Sigma-Aldrich, St. Louis, MO, USA) from 0.3 g of berry powder following the manufacturer’s instructions. The quality and concentration of RNA were detected by agarose gel electrophoresis and using a NanoDrop 2000 (Thermo Fisher Scientific, Waltham, MA, USA). The RNA-seq analysis was performed by IGA Tech (IGA Technology Services Srl, Udine, Italy), sequencing the libraries on paired-end 150-bp mode on NovaSeq 6000 (Illumina, San Diego, CA, USA), including base calling and demultiplexing, adapters masking, trimming, alignments on the reference genome (12xCHR v2.1), quality control (statistics on strandness of reads, on gene body coverage, on read distribution, and on insert size), and pairwise differential expression analysis. Functional annotations of genes were retrieved from the Integrape grapevine reference catalog (Navarro-Payà et al., 2021).

### Experimental design and statistical analysis

Vines were arranged in three rows. Each row included the six irrigation treatments (eight vines for each irrigation regime) for a total of 24 vines per irrigation regime. Significant differences between treatments were determined by one-way analysis of variance (ANOVA, *P* ≤ 0.05). Means of irrigation treatments were separated by Tukey’s HSD. All statistical analysis and the heatmaps representing log2 fold change (log2FC) of metabolites concentrations between treatments (RDI/FI) were performed using JMP Pro 16.1 (SAS Institute Inc., Drive Cary, NC, USA).

### Accession number

All raw sequence reads have been deposited in NCBI Sequence Read Archive (https://www.ncbi.nlm.nih.gov/sra). The BioProject accession is PRJNA886074.

## Results

### Climatic conditions and phenology

Annual precipitations (970 and 1,060 mm in 2019 and 2020, respectively), reference evapotranspiration (900 and 891 mm, respectively), and annual mean air temperature (16.0°C and 15.8°C, respectively) were quite similar between years (**Figure S1**). However, the mean air temperature in April and May was lower in 2019 (14.7°C, average of the 2 months) than in 2020 (16.5°C), resulting in a 7-day shift of fruit set and in turn of the berry pea-size stage (**Table 1**; **Figure S1**). Consequently, the average veraison and harvest dates were also slightly different between years (**Table 1**). Water deficit modulated vine physiology as well, delaying veraison in RDI-1 plants and affecting the harvest date in all treatments (**Table 1**).

### Vine water status and vegetative growth

**Figure 1:**
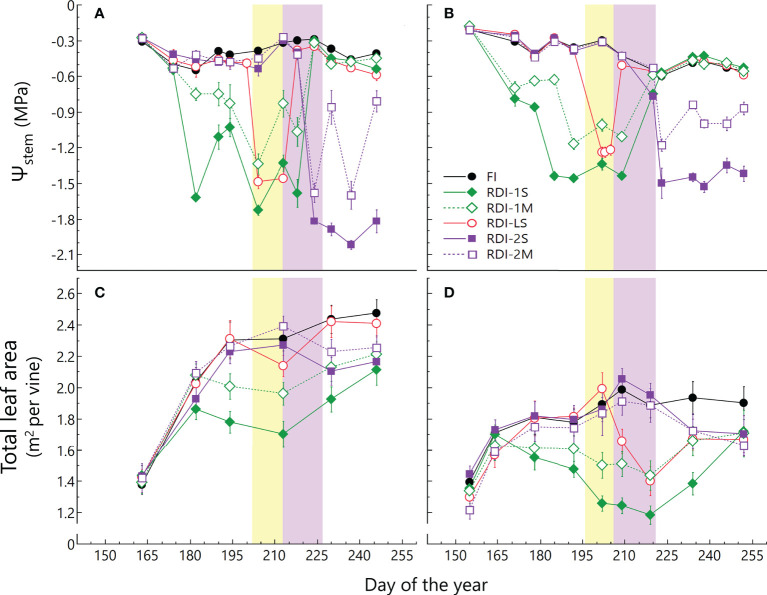
Seasonal course of stem water potential (*Ψ*_stem_) and total leaf area per vine measured in 2019 **(A, C)** and 2020 **(B, D)** in Sangiovese grapevines (*Vitis vinifera* L.) subjected to six different irrigation regimes (FI, full irrigation from pea-size berry to harvest; RDI-1S and RDI-1M, severe and moderate water deficit applied from pea-size berry to veraison; RDI-LS, water deficit applied during the lag-phase; RDI-2S and RDI-2M, severe and moderate water deficit applied from veraison to harvest). Values are means ± standard deviation of five vines per treatment. The yellow and violet boxes indicate the lag-phase and veraison period, respectively.

The seasonal course of *Ψ*_stem_ was consistent with irrigation volumes supplied in both years (**Figure 1B**). The *Ψ*_stem_ of FI vines ranged between −0.30 and −0.55 MPa in 2019 and between −0.20 and −0.60 MPa in 2020. During the pre-veraison period, the lowest values of *Ψ*_stem_ were measured for RDI-1S vines (−1.73 and −1.46 MPa in 2019 and 2020, respectively), whereas after veraison, the lowest values of *Ψ*_stem_ were measured in RDI-2S vines (−2.00 and −1.53 MPa in 2019 and 2020, respectively). The RDI-LS vines experienced water deficit only during the lag-phase, reaching minimum *Ψ*_stem_ values of −1.49 and −1.24 MPa in 2019 and 2020, respectively. The TLA per vine was higher in 2019 than in 2020 (+19%), due to the more favorable climatic conditions for vegetative growth that occurred in spring 2019 (**Figure 1D**; **Figure S1**). The TLA of all RDI treatments readily responded to the lower water availability showing significant differences between irrigation treatments starting from 19 days (DOY 182, 2019) and 16 days (DOY 172, 2020) after the irrigation differentiation (**Figure 1D**). In FI vines, TLA showed an increment until veraison and then it remained quite stable (an average of 2.00 and 1.74 m^2^ per vine in 2019 and 2020, respectively) until harvest. The greatest impact on canopy growth was measured when water deficit was applied from berry pea-size (RDI-1 treatments) due to basal leaf abscission (visually assessed) and a contemporary reduction in vegetative growth. In particular, RDI-1S vines showed the lowest average values of TLA during the entire growing season (1.70 and 1.40 m^2^ in 2019 and 2020, respectively). The TLA reduction induced in post-veraison stressed vines (RDI-2 treatments) was less pronounced (−16% than FI vines, average between years and treatments) than that measured in RDI-1 vines (**Figure 1**), and it was mainly due to basal leaf fall. A reduction in leaf area was also observed in RDI-LS vines at DOY 213 (2019) and 209 (2020) due to the water stress imposed only during the lag-phase. However, new leaves promptly developed after the stress period, leading to similar TLA values after veraison in RDI-LS and FI vines in 2019.

### Cluster temperature and light exposure

**Figure 2:**
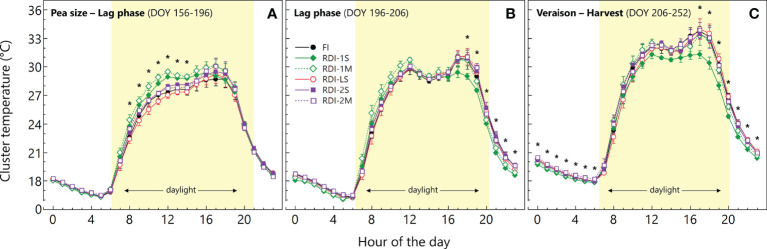
Daily courses of cluster temperature measured in the 2020 growing season in Sangiovese grapevines (*Vitis vinifera* L.) subjected to six irrigation regimes FI, full irrigation from pea-size berry to harvest; RDI-1S and RDI-1M, severe and moderate water deficit applied from pea-size berry to veraison; RDI-LS, water deficit applied during the lag-phase; RDI-2S and RDI-2M, severe and moderate water deficit applied from veraison to harvest). The daily courses are the average of DOY 156–196 **(A)**, DOY 196–206 **(B)**, and DOY 206–252 **(C)**. Symbols are hourly mean values ± standard deviation of three replicates per treatment. Asterisks indicate significant differences between irrigation treatments after analysis of variance (ANOVA) within each hour (*P* < 0.05).

**Figure 3:**
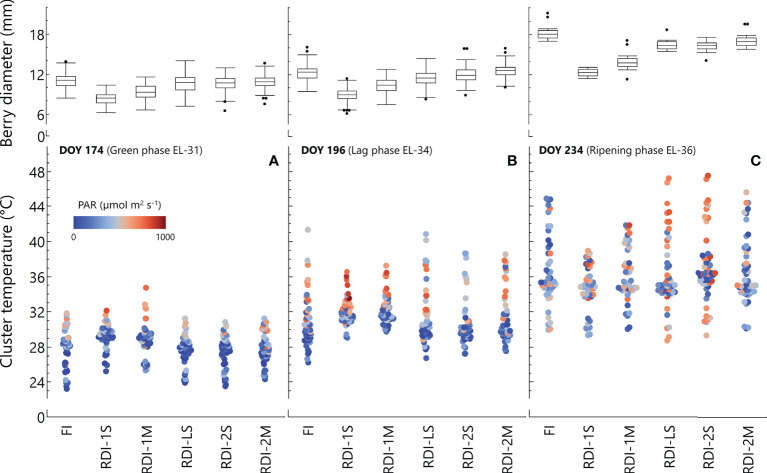
Cluster intercepted photosynthetic active radiation (PAR), cluster temperature, and berry diameter were simultaneously measured at three different dates [green phase, DOY 174 **(A)**; lag-phase, DOY 196 **(B)**; ripening phase, DOY 234 **(C)**] in 2020 in Sangiovese grapevines (*Vitis vinifera* L.) subjected to six different irrigation regimes (FI, full irrigation from pea-size berry to harvest; RDI-1S and RDI-1M, severe and moderate water deficit applied from pea-size berry to veraison; RDI-LS, water deficit applied during the lag-phase; RDI-2S and RDI-2M, severe and moderate water deficit applied from veraison to harvest). Berry diameters are means ± standard deviations of 81 berries per irrigation treatment.

The average cluster temperature from berry pea-size to the beginning of the lag-phase showed significantly higher values in both RDI-1 treatments between 08:00 and 14:00 (**Figure 2**). At the same stage (DOY 174), the daily measurements revealed high light exposure in RDI-1 clusters (**Figure 3**), associated with higher temperatures especially in RDI-1M clusters which had bigger berries with respect to RDI-1S ones (8.9 and 8.2 mm of berry diameter, respectively). During the lag-phase, the average cluster temperature was significantly different in the late afternoon and night (**Figure 2**), and in particular, RDI-1S clusters were cooler (−2.0°C) from 18:00 to 23:00 with respect to the other treatments. The daily patterns during the lag-phase (DOY 196) reported in **Figure 3** showed that, despite the higher light exposure, the cluster temperature in both RDI-1 regimes did not reach the upper values measured in clusters of the other treatments. Indeed, berries from RDI-1 vines were significantly smaller than those sampled from FI and RDI-2 vines (**Figure 3**). During ripening, the RDI-1S clusters had significantly lower temperatures from 17:00 to 06:00 with respect to those measured in clusters from other treatments, remaining cooler all the night (**Figure 2**). On DOY 234, RDI-1S clusters maintained lower maximum temperature throughout the day (39.4°C) than in clusters from other treatments (**Figure 3**). The RDI-LS and RDI-2S clusters showed higher light exposure and reached high temperatures (47.2°C and 47.5°C, respectively). The RDI-1S cluster had smaller berries characterized by a lower diameter, thus reducing cluster compactness and improving air circulation (**Figure 3**).

### Yield parameters and berry characteristics

Irrigation significantly affected yield and berry parameters at harvest, with the only exception of cluster weight in 2019 (**Table 2**). Yields ranged between 2,199 and 3,161 g per vine and 1,831 and 3,232 g per vine in 2019 and 2020, respectively, with the lowest and highest values measured in RDI-1S and FI vines, respectively (**Table 2**). Cluster weight and berry FW were consistent with the volume of water supplied by irrigation. Berry FW ranged from 1.48 g (RDI-1S) to 2.54 g (FI), and it was strongly affected by pre-veraison water deficit. The relative skin mass was higher in RDI-2S berries (19.03% and 17.24%, in 2019 and 2020, respectively) even if it showed significant differences in both years only with respect to FI berries, which had the lowest values (**Table 2**). The TSS were similar between treatments in both years since fruits were harvested once the threshold of 22 ± 0.5°Brix was reached (**Table 2**). The highest and lowest values of pH and TA, respectively, were measured in the juice from the berries of RDI-1S vines in both years (**Table 2**), whereas RDI-LS berries had the highest level of TA at harvest (6.37 and 6.90 g L^−1^ of tartrate, in 2019 and 2020, respectively). No significant differences in TA were measured between RDI-LS and FI berries (**Table 2**)

**Table 2:** Yield (FW), cluster weight (FW), berry fresh weight (FW), relative skin mass, yield-to-pruning weight ratio, total soluble solid (TSS), pH, and titratable acidity (TA) of juice measured in 2019 and 2020 in Sangiovese grapevines (*Vitis vinifera* L.) subjected to six different irrigation regimes.

Year	Irrigation treatment	Yield (g per vine)	Cluster weight (g)	Berry FW (g)	Relative skin mass (%)	Yield-to-pruning weight ratio	TSS(°Brix)	pH	TA (g L^−1^ tartrate)
2019	FI	3,161 a	363	2.53 a	14.51 c	4.51 a	22.4	3.56 ab	6.27 a
	RDI-1S	2,199 b	287	1.47 c	17.63 ab	3.60 c	22	3.62 a	5.00 c
	RDI-1M	2,598 ab	324	1.78 bc	14.55 c	3.88 bc	22.7	3.44 bc	5.30 bc
	RDI-LS	2,805 ab	351	2.13 ab	15.36 bc	4.06 ab	21.91	3.42 c	6.37 a
	RDI-2S	2,896 ab	325	2.30 a	19.03 a	4.33 ab	21.8	3.49 bc	5.90 ab
	RDI-2M	3,068 a	355	2.41 a	16.92 abc	4.65 a	22.2	3.48 bc	5.79 abc
	*HSD*	850	n.s.	0.5	1.88	0.73	n.s.	0.13	0.86
2020	FI	3,232 a	329 a	2.54 a	13.18 b	4.99 a	22.73	3.34 b	6.75 a
	RDI-1S	1,831 c	210 b	1.48 d	16.22 ab	2.41 c	22.37	3.58 a	5.28 b
	RDI-1M	2,125 ab	241 b	1.72 cd	15.57 ab	3.23 bc	22.1	3.37 b	5.65 b
	RDI-LS	2,604 ab	281 ab	2.05 bc	14.00 ab	3.83 ab	22.13	3.38 b	6.90 a
	RDI-2S	2,414 ab	255 ab	2.13 b	17.24 a	3.44 bc	21.9	3.47 ab	6.58 a
	RDI-2M	2,431 ab	265 ab	2.23 ab	15.84 ab	3.79 ab	22.6	3.41 b	6.60 a
	*HSD*	709	84.31	0.34	2.76	1.64	n.s.	0.15	0.7


Values are means of three replicates per treatment. Different letters indicate honest significant differences (HSD) between irrigation treatments after analysis of variance (ANOVA) within each year.

n.s., not significant.

### Flavonoid accumulation

**Figure 4:**
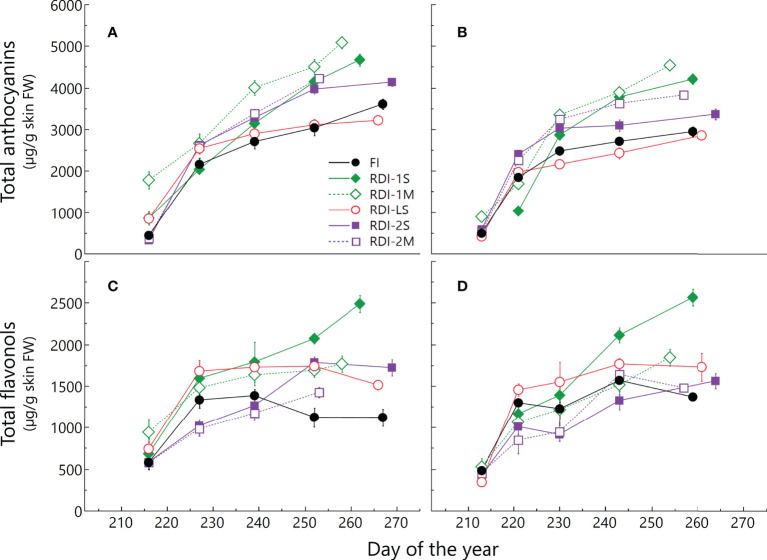
Total anthocyanin and total flavonol accumulation from veraison to harvest in 2019 **(A, C)** and 2020 **(B, D)** in berries of Sangiovese grapevines (*Vitis vinifera L*.) subjected to six different irrigation regimes (FI, full irrigation from pea-size berry to harvest; RDI-1S and RDI-1M, severe and moderate water deficit applied from pea-size berry to veraison; RDI-LS, water deficit applied during the lag-phase; RDI-2S and RDI-2M, severe and moderate water deficit applied from veraison to harvest) in 2019 and 2020. Values are means ± standard deviations of three replicates per irrigation treatment.

In both years, irrigation affected skin flavonoid accumulation during ripening. The total anthocyanin content increased rapidly in all the treatments until DOY 239 (2019) and 230 (2020). After these dates, differences between treatments were more pronounced: berries from RDI-1 vines still strongly accumulated anthocyanins until harvest, whereas FI and RDI-LS berries showed a lower increment rate (**Figures 4A, B** and **Table S2**). Intermediate accumulation rates were measured in berries of both RDI-2 treatments. At harvest, berries sampled from both RDI-1 vines showed the highest anthocyanin concentration. In particular, RDI-1M berries had 5,083 and 4,551 µg of anthocyanins per gram of skin FW in 2019 and 2020, respectively (**Table 3**). Post-veraison water deficit increased anthocyanin content as well, whereas the lowest values were measured in RDI-LS berries (−11% and −3% than FI, in 2019 and 2020, respectively). In both years, cyanidin glucoside was the most abundant in FI, RDI-LS, RDI-1S, and RDI-1M berries, whereas malvidin glucoside reached the highest concentration in berries from both RDI-2 treatments (**Table 3**). Significantly higher values were measured in RDI-1M for the single non-acylated anthocyanidins and for their sum as well. Both RDI-1 berries showed the highest hydroxylated, methoxylated, tri- and di-substituted anthocyanin concentrations. The tri- over di-substituted ratio was significantly higher in both RDI-2 berries as a consequence of the higher amount of tri-hydroxylated forms (**Table 3**). Skin total flavonol increment was strongly enhanced in both years by RDI-1S irrigation treatments (**Figures 4C, D**, **Table S3**). The highest content of flavonols at harvest was measured in berry skin from severe pre-veraison water-stressed vines than those FI ones (+126% and +84%, in 2019 and 2020, respectively), which in turn showed the lowest values (1,120 and 1,369 µg g^−1^ skin FW in 2019 and 2020, respectively). Quercetin’s derivative compounds were the most abundant within flavonols (**Table 4**). In particular, quercetin glucoside represented 52% of the total flavonols and was enhanced in RDI-1S berries.

**Table 3:** Anthocyanin composition measured at harvest in the berries of Sangiovese grapevines (*Vitis vinifera* L.) subjected to six irrigation regimes in 2019 and 2020.

	2019							2020							
	FI	RDI-1S	RDI-1M	RDI-LS	RDI-2S	RDI-2M	*I*	FI	RDI-1S	RDI-1M	RDI-LS	RDI-2S	RDI-2M	*I*
Delphinidin-GS	348 c	462 ab	520 a	243 d	342 c	418 b	***	260 bc	467 a	525 a	217 c	280 b	309 b	***
Cyanidin-GS	1176 bc	1386 ab	1618 a	1059 c	1032 c	1149 bc	**	1062 b	1332 a	1380 a	951 bc	824 c	909 c	***
Petunidin-GS	374 d	513 b	620 a	297 e	418 cd	457 bc	***	299 b	562 a	604 a	279 b	354 b	395 b	***
Peonidin-GS	657 c	1077 a	1035 ab	609 c	929 b	923 b	***	444 b	724 a	824 a	495 b	713 a	785 a	***
Malvidin-GS	993 bc	1171 ab	1229 a	957 c	1363 a	1224 a	**	833 c	1060 b	1188 b	855 c	1145 b	1381 a	**
Peonidin-Ace-GS	1.8	2.6	2.6	2.3	1.8	2.1	n.s.	3.0	3.3	2.2	2.6	2.1	2.0	n.s.
Malvidin-Ace-GS	4.4	3.3	3.6	2.5	3.0	3.1	n.s.	2.1 b	2.9 a	2.0 b	2.4 ab	2.4 ab	2.4 ab	n.s.
Peonidin-Caf-GS	2.9 ab	2.3 b	4.5 a	3.4 ab	4.2 a	3.3 ab	*	3.0 b	4.4 a	2.0 c	3.0 b	3.2 b	3.4 b	***
Malvidin-Caf-GS	1.9 a	1.0 b	1.4 ab	1.4 ab	1.8 ab	1.2 ab	n.s.	1.0 c	1.6 ab	2.0 a	1.1 bc	1.2 bc	1.2 bc	n.s.
Cyanidin-Cou-GS	23 a	24 a	23 a	17 a	12 b	17 a	**	15 ab	21 a	2.3 c	20 a	13 b	13 b	***
Petunidin-Cou-GS	2.2 ab	2.1 b	3.1 a	2.8 ab	2.5 ab	2.6 ab	n.s.	3.8 ab	4.3 a	2.0 b	3.5 ab	3.1 ab	2.9 ab	*
Peonidin-Cou-GS	14 ab	16 a	12 b	8.7 c	11 bc	11 bc	**	7.8	11	8.1	9.9	9.1	9.8	n.s.
Malvidin-Cou-GS	8.4 ab	7.7 b	11 ab	11 ab	12 a	10 ab	n.s.	11	13	9.1	12	12	12	n.s.
3'4' OH	1875 c	2508 a	2695 a	1699 d	1990 bc	2105 b	***	1535 c	2096 a	2219 a	1482 c	1564 c	1722 b	***
3'4'5' OH	1732 c	2160 b	2388 a	1515 c	2142 b	2116 b	***	1410 c	2111 a	2332 a	1370 c	1798 b	2104 a	***
3'4'5'/3'4	0.92 b	0.86 b	0.89 b	0.89 b	1.08 a	1.00 ab	***	0.92 b	1.01 ab	1.05 a	0.92 b	1.15 a	1.22 a	**
OH- sub forms	1547 b	1872 ab	2161 a	1319 c	1386 c	1584 b	***	1337 c	1820 b	1907 a	1188 d	1117 d	1231 cd	***
OCH3- sub forms	2060 b	2796 a	2922 a	1895 b	2746 a	2637 a	***	1608 c	2387 ab	2643 a	1664 c	2245 b	2595 a	***
Σ acylated	59 a	59 a	61 a	49 b	48 b	50 b	**	47 ab	62 a	30 c	55 ab	46 bc	47 bc	***
Σ not-acylated	3548 d	4609 b	5022 a	3165 e	4084 c	4171 c	***	2898 e	4145 b	4521 a	2797 e	3316 d	3779 c	***
Total anthocyanins	3607 d	4668 b	5083a	3214 e	4132 c	4221 c	***	2945 de	4207 b	4551 a	2852 e	3362 d	3826 c	***

Values (µg g^−1^ of skin fresh weight) are the means of three replicates for each irrigation treatment. Different letters indicate significant differences between irrigation treatments after analysis of variance (ANOVA) and Tukey’s HSD test within each year. P-values are indicated: *, P < 0.05; **, P < 0.01; ***, P < 0.001; n.s., not significant.

GS, glucoside; Ace, acetyl; Caf, caffeoyl; Cou, coumaroyl.

**Table 4:** Flavonols composition measured at harvest in berries of Sangiovese grapevines (*Vitis vinifera* L.) subjected to six irrigation regimes in 2019 and 2020.

	2019							2020							
	FI	RDI-1S	RDI-1M	RDI-LS	RDI-2S	RDI-2M	*I*	FI	RDI-1S	RDI-1M	RDI-LS	RDI-2S	RDI-2M	*I*
Myricetin-GN	57 d	100 a	80 abc	67 cd	91 ab	72 bcd	**	99	138	137	105	104	89	ns
Myricetin-GS	59 c	121 a	72 bc	66 c	120 ab	80 abc	**	65 c	113 a	84 b	65 c	95 ab	87 b	**
Quercetin-GN	109 c	233 a	137 bc	125 c	225 ab	167 abc	**	130 c	210 a	219 a	122 c	175 ab	159 bc	***
Quercetin-GA	217 d	547 a	414 b	289 c	300 c	267 cd	***	265 b	480 a	274 b	338 b	254 b	251 b	***
Quercetin-GS	595 d	1381 a	1000 b	809 bc	833 bc	720 cd	***	699 c	1312 a	883 b	935 b	786 bc	755 c	***
Quercetin-RT	16 d	28 a	22 bc	21 bcd	24 ab	18 cd	**	13	24	21	19	23	19	ns
Kaempferol-GA	8.8	25	15	19	17	15	ns	17 c	37 a	28 ab	22 bc	18 bc	18 bc	**
Kaempferol-GN	4.2	13	8.7	7.4	7.7	7.0	ns	5.3	9.5	11	7.8	5.9	6.1	ns
Kaempferol-GS	36 d	105 a	65 c	90 ab	69 bc	56 cd	***	53 c	143 a	101 b	86 bc	67 bc	67 bc	***
Isorhamnetin-GA	2.1	4.3	3.7	1.9	3.4	2.4	ns	6.7	8.1	7.5	5.6	5.7	4.9	ns
Isorhamnetin-GN	11	22	20	14	21	15	ns	11	22	17	18	21	17	ns
Isorhamnetin-GS	4.5	6.8	4.6	4.9	9.7	4.9	ns	5.3	6.9	6.3	6.0	8.3	6.4	ns
Total flavonols	1120 e	2484 a	1800 b	1514 cd	1721 bc	1424 d	***	1369 c	2561 a	1847 b	1729 bc	1563 bc	1479 bc	***

Values (µg g^-1^ of skin fresh weight) are means of three replicates. Different letters indicate significant differences between irrigation treatments after analysis of variance (ANOVA) and Tukey’s HSD test within each year. P-values are indicated: *, P<0.05; **, P<0.01; ***, P<0.001; n.s., not significant. GS, glucoside; GN, glucuronide; GA, galactoside; RT, rutinoside.

### Phenylpropanoid and flavonoid pathways

**Figure 5:**
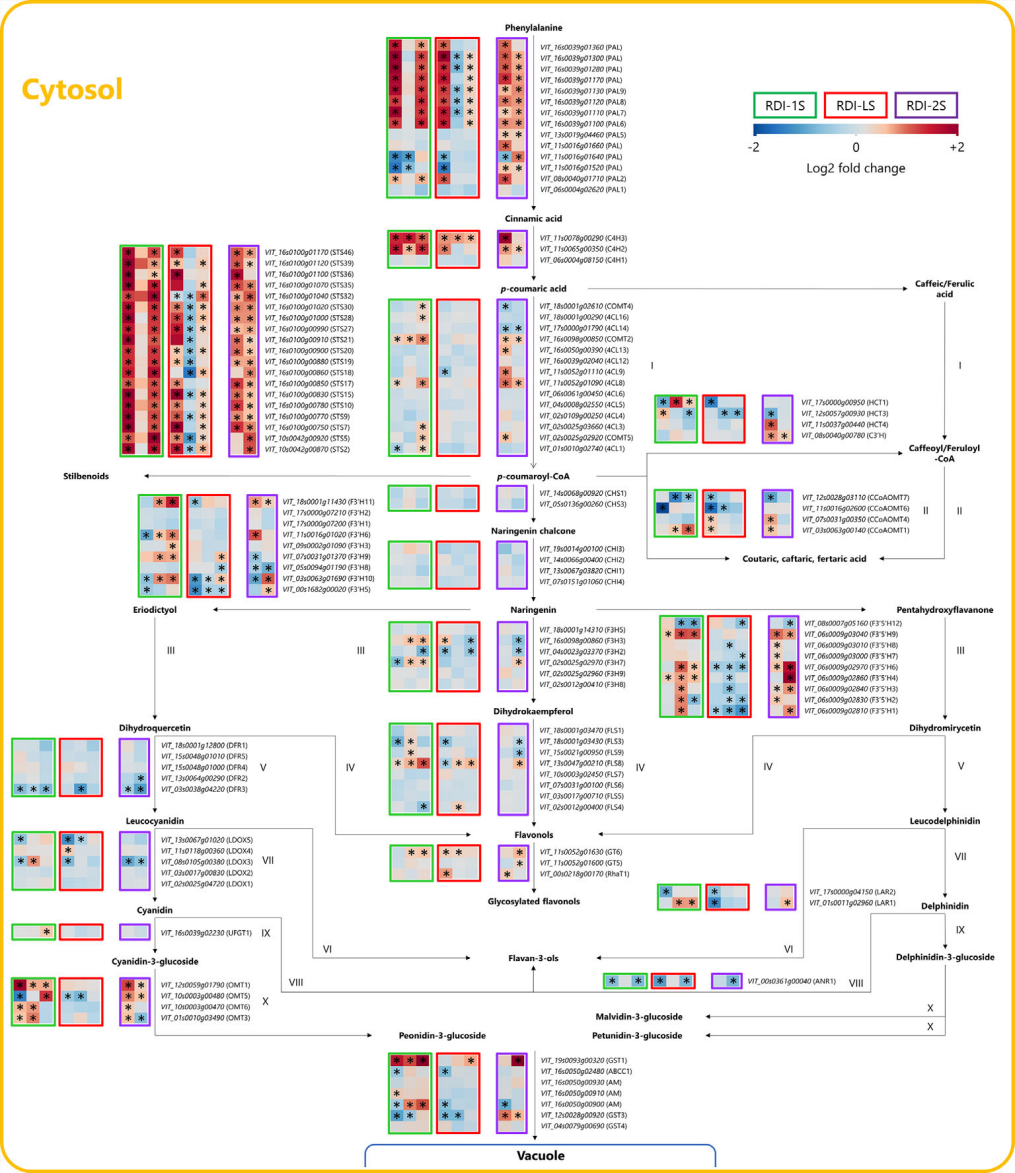
Modulation of phenylpropanoid and flavonoid pathways in berries of Sangiovese grapevines (*Vitis vinifera* L.) subjected to different irrigation regimes: RDI-1S, severe water deficit applied from pea-size berry to veraison (green block); RDI-LS, water deficit applied during the lag-phase (red block); and RDI-2S, severe water deficit applied from veraison to harvest (purple block). Log2FC (RDI/FI) levels of differential gene expression are presented at the beginning of veraison (left box), mid-ripening (central box), and harvest (right box). Asterisks identify significant differences (*P* < 0.05) between treatments. Roman numbers identify commonly regulated steps of the pathway.

**Figure 6:**
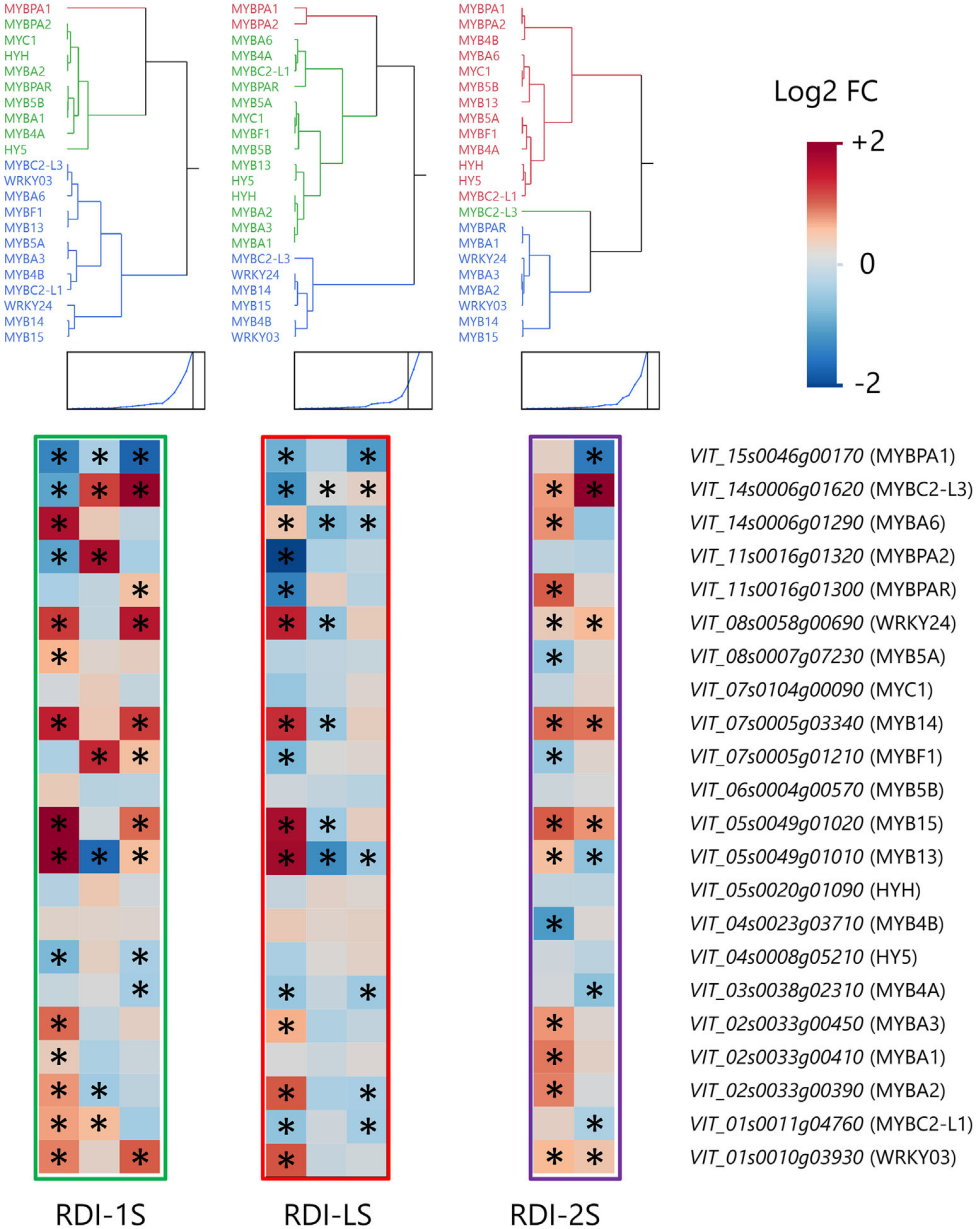
Modulation of transcription factors of the phenylpropanoid and flavonoid pathways in berries of Sangiovese grapevines (*Vitis vinifera* L.) subjected to different irrigation regimes: RDI-1S, severe water deficit applied from pea-size berry to veraison (green block); RDI-LS, water deficit applied during the lag-phase (red block); and RDI-2S, severe water deficit applied from veraison to harvest (purple block). Log2FC (RDI/FI) levels of differential gene expression are presented at the beginning of veraison (left box), mid-ripening (central box), and harvest (right box). Asterisks identify significant differences (*P* < 0.05) between treatments. Genes were hierarchically clustered based on their response to water deficit.

Irrigation regimes modulated the expression of many genes of the phenylpropanoid and flavonoid pathways at the three sampling dates (**Figure 5**). Seven phenylalanine ammonia lyases (*PALs*) were upregulated at veraison and harvest by RDI-1S and RDI-LS and at mid ripening and harvest by RDI-2S. The highest expression of two cinnamate-4-hydroxylases (*C4H2 and C4H3*) was measured at all sampling dates in RDI-1S berries. All the 19 stilbene synthases (*STS*) detected were overexpressed under RDI-1S conditions at veraison and harvest, and most of them also showed an overexpression under RDI-2S at mid ripening and harvest. In the flavonoid pathway, the two most modulated genes were *F3′Hs* and *F3′5′Hs*. The RDI-1S significantly promoted the expression of both types of hydroxylases particularly at mid ripening and harvest. In RDI-2S berries, *F3′5′H* genes were more upregulated than *F3′H* (particularly *F3′5′H4* and *F3′5′H6* at harvest). The expression of the flavonoid-3-*O*-glucosyltransferase (*UFGT1*) for the biosynthesis of cyanidin- and delphinidin-3-glucoside was promoted by RDI-1S at harvest. The *O*-methyltransferases (*OMTs*) were overexpressed by RDI-1S and RDI-2S for the biosynthesis of malvidin-, petunidin-, and peonidin-3-glucoside, whereas *OMT5* was downregulated at veraison and mid ripening in RDI-LS berries. Glutathione *S*-transferase 1 (*GST1*) was particularly overexpressed by RDI-1S in all the sampling dates, whereas a *MATE*-type transporter (*AM*) and the *GST3* were upregulated at mid ripening and harvest by RDI-1S and RDI-2S, respectively, but downregulated by RDI-LS at veraison. The expression of flavonol synthase 8 (*FLS8*) was significantly promoted by all irrigation treatments, particularly RDI-1S at all sampling dates. Similarly, flavonol glycosylation was induced under all RDI regimes through the upregulation of flavonol-3-*O*-glycosyltransferases (*GT5* and *GT6*) and flavonol-3-*O*-rhamnosyltransferases (*RhaT1*). The transcription factors *MYBA2, MYBA3*, and *MYBA6* were overexpressed in RDI-1S and RDI-LS berries at veraison and in RDI-2S at mid ripening (**Figure 6**). Similar results were observed for *MYB13, MYB14, MYB15, WRKY03,* and *WRKY24*, in accordance with *STSs* expression (**Figure 6**). *MYBF1*, related to the *FLSs*, was upregulated by RDI-1S at mid ripening and harvest, but it was not in RDI-2S and RDI-LS berries.

## Discussion

The results of the current study produced novel evidence about the following: i) the effect of timing and intensity of water stress on berry flavonoid accumulation, ii) the possible collateral effects of light and temperature on the accumulation of these compounds in berry, and iii) the modulation of the biosynthetic pathways of anthocyanins and flavonols in berries under different water availability conditions.

Regarding the period when the water stress was applied, either RDI-1 or RDI-2 treatments induced higher anthocyanin and flavonol concentrations than FI berries, but the water deficit imposed before veraison had a greater effect to enhance their biosynthesis and accumulation. In both years, characterized by different climatic conditions, the berries from vines subjected to water deficit from berry pea-size to veraison (RDI-1) showed the highest total anthocyanin concentration at harvest. The pattern observed for total anthocyanin was similar to those of many non-acylated anthocyanin (and their sum), as well as for hydroxylated, methoxylated, and tri- and di-substituted forms, highlighting a broad triggering effect of the water deficit before veraison on the abovementioned compounds. Berries from RDI-2 vines showed intermediate values of berry anthocyanins with respect to those measured in FI and RDI-1 berries. In particular, the anthocyanin profile was characterized by the highest content of malvidin-3-glucoside and, as a consequence, of the tri- over di-substituted ratio. Previous studies already reported a positive effect of water deficit on total anthocyanins, especially when it was applied before veraison. Caruso et al. (2022) observed higher anthocyanin content in pre-veraison water-stressed Sangiovese grapevines with respect to post-veraison stressed vines. Similarly, Palliotti et al. (2014) reported that water deficit imposed before veraison increased berry anthocyanin concentration with respect to full irrigation. Similar results were also reported for other cultivars (Intrigliolo et al., 2012; Shellie, 2014) although a greater accumulation induced by post-veraison water deficit was also measured (Girona et al., 2009; Basile et al., 2011; Ollè et al., 2011). Despite many studies reporting no effect of water stress on flavonol accumulation (Martínez-Lüscher et al., 2014; Yu et al., 2016; Savoi et al., 2017), we observed that pre-veraison water deficit increased its concentration. According to our results, in pre-veraison water-stressed Shiraz vines, a significant increment in flavonol accumulation was reported (Ojeda et al., 2002). Moreover, in sustained deficit irrigated Chardonnay and Tocai Friulano grapevines, a significantly higher content of berry flavonols was observed at harvest with a transient increase in the expression of the flavonol synthases during ripening (Deluc et al., 2009; Savoi et al., 2016).

The severity of water stress also played an important role in flavonoid accumulation, either in RDI-1 and RDI-2 treatments. In particular, the highest concentration of anthocyanins and flavonols was measured under moderate and severe water stress, respectively. The higher anthocyanin accumulation rate and final concentration observed in the berries from moderate RDI-2 stressed vines could be related to the higher total leaf area and/or to the greater photosynthetic rate (data not shown), allowing to accelerate berry ripening and anthocyanin accumulation with respect to severely stressed vines (Herrera and Castellarin, 2016). Similarly, the moderately stressed vines before veraison took advantage of the greater TLA (**Figure 1**) and photosynthetic rates (data not shown) with respect to the severely stressed ones, also supporting a faster post-veraison recovery from water stress. On the contrary, berry skin flavonols at harvest were higher in berries sampled from severely stressed RDI-1 vines in both years. This different response of anthocyanins and flavonols to the level of water stress can also be explained by the different impacts of light and temperature on these compounds.

We monitored cluster temperature and cluster light exposure in order to discriminate their impact on anthocyanin and flavonol accumulation from the effects induced by water stress. Regarding temperature, clusters of RDI-1S vines were the only ones at significantly lower temperatures during the lag and ripening phases (**Figure 2**) due to the smaller berries and lower cluster compactness, however, the highest berry anthocyanin concentration was measured in RDI-1M vines, which had cluster temperatures similar to those from the other irrigation treatments. Regarding sunlight exposure, clusters of RDI-1 vines were more exposed during the lag-phase due to the leaf abscission caused by water deficit (**Figure 3**). However, during ripening, the incident radiation on clusters was similar within all treatments subjected to water stress, not allowing to draw a direct correlation with anthocyanin accumulation. For example, RDI-2M berries had a higher anthocyanin content than RDI-2S but had a lower cluster light exposure. Similarly, RDI-LS berries showed low anthocyanin content even if sampled from clusters well exposed to sunlight (**Figure 3**). Thus, although RDI treatments modified cluster sunlight exposure and temperature, which are key factors to modulate anthocyanin biosynthesis (Mori et al., 2005; Tarara et al., 2008; Blancquaert et al., 2019; Yan et al., 2020), our data suggest a predominant effect of water stress as the main driver for the anthocyanin accumulation in deficit irrigated vines. On the contrary, the lower and higher flavonol concentrations measured at harvest in FI and RDI-1S berries, respectively (sampled from the less and the more illuminated clusters, respectively), seem to confirm the role of light as the main factor in the regulation of flavonol biosynthesis and accumulation (Spayd et al., 2002; Martínez-Lüscher et al., 2014; Friedel et al., 2016). During the lag-phase, clusters from RDI-1 vines were more exposed to light with respect to the other treatments, but only RDI-1S berries had greater flavonol increment and final concentration. This evidence suggests the interaction of a further factor beyond light exposure which could be represented by berry temperature. Indeed, only in RDI-1S vines, since the lag-phase, the cluster temperature was significantly lower than that measured in clusters from the other treatments, taking advantage of the small berry size (**Figure 2**). The measurements taken during ripening (**Figures 2**, **3C**) showed that while the light environment was similar, the smaller RDI-1S berries were cooler, especially during the late afternoon and night. Thus, we hypothesize that light was the main driver for the flavonol biosynthesis until the berry temperature became too high and limiting. It is worth noting that all treatments, except RDI-1S, showed consistent flavonol accumulation patterns between the two experimental years, keeping below about 1,750 µg/g skin FW (**Figures 4C** **)**. It cannot be excluded that, at our experimental conditions, this value represented a sort of threshold coincided with the ripening moment on which cluster compactness determines the thermal upper limit for the flavonol biosynthesis. These results agree with what was found by Yan et al. (2020), who compared the effect of different day/night thermal regimes on flavonol accumulation. They reported that high temperatures inhibited flavonol accumulation mainly downregulating the *FLS* genes at the transcriptional level. Even in a field experiment, Martínez-Lüscher et al. (2019) hypothesized that high temperature depressed the biosynthesis of berry flavonols of Merlot grapevine.

The effect of water shortage is also directly related to specific modulation of the flavonoid biosynthetic pathway. Castellarin et al. (2007) reported that an early water deficit upregulated the anthocyanin biosynthesis in Cabernet Sauvignon berries, inducing a greater and earlier expression of many genes of the flavonoid biosynthetic pathway. In particular, they reported that *F3′Hs* and *F3′5′Hs* were the key modulated genes through the pathway and *F3′5′Hs* were significantly upregulated in berries from early water-stressed vines. Similar findings especially related to *PAL*, *C4H*, *4CL*, *CHS*, *F3′5′H*, *LDOX*, and *UFGT* were reported for berries of the same cultivar from deficit irrigated (30% ET_c_) vines until 30 days prior to harvest (Yang et al., 2020). We showed that the biosynthetic steps corresponding to the genes *F3′Hs*, *F3′5′Hs*, *F3Hs*, *OMTs*, and *GSTs* were the most regulated, particularly overexpressed in RDI-1S berries, even far after water deficit was released. It is interesting to note that in berries from pre-veraison water-stressed vines, the *F3′H* hydroxylases were upregulated since mid ripening causing the lowest tri- over di-substituted ratio measured at harvest (**Table 3**). This overregulation of the di-hydroxylated branch of the pathway was not observed previously (Castellarin et al., 2007; Savoi et al., 2017; Yang et al., 2020) and could represent a specific modulation of Sangiovese and other cultivars characterized by a prevalence of di-substituted forms in the lateral ring (Mattivi et al., 2006; Squadrito et al., 2010). On the contrary, post-veraison water deficit particularly upregulated many genes involved in the tri-hydroxylation of the B ring and only a few responsible for the di-hydroxylation. These results explain the higher tri- over di-substituted ratio at harvest which characterized berries from both the RDI-2 treatments and the high concentration of malvidin-3-glucoside (**Table 3** and **Table S2**). Some authors observed a higher concentration of malvidin-3-glucoside and, often, a lower accumulation of cyanidin-3-glucoside in berries from vines of cv. Shiraz and Merlot subjected to post-veraison water deficit (Ollè et al., 2011; Herrera et al., 2017). From an oenological point of view, the highest levels of tri-/di-substituted ratio observed in RDI-2 berries shift the pigment hue to more purple–blue and could be particularly important for Sangiovese which often is characterized by low pigment content. We observed a significant upregulation of the expression of *OMTs* during ripening, far after the water deficit was released, suggesting that these were a further key point of regulation under water deficit conditions (Ju et al., 2019; Guo et al., 2021). Moreover, *GST1* and *AM* were strongly upregulated by RDI-1S, leading to speculate a greater and earlier storage rate of anthocyanins. The low anthocyanin concentrations measured at harvest in RDI-LS were clearly related to the downregulation of many genes through the pathway, and further investigations are needed to deepen this effect. Among TFs related to the flavonoid pathway, our data support the hypothesis of a correlation between *MYBA1–2* and *F3′5′Hs* during berry ripening (Rinaldo et al., 2015; Savoi et al., 2017) as well as the capacity of MYBA6 to influence the anthocyanin accumulation and composition under severe environmental conditions (Czemmel et al., 2017). It is interesting to note that either *MYBC2-L1* or *MYBC2-L3*, a repressor of anthocyanin biosynthesis (Zhu et al., 2018), was overexpressed in berries from RDI-1S vines which had a higher anthocyanin content and was downregulated in RDI-LS berries which had a lower anthocyanin content. Similar results were observed by Xie et al. (2020) who proposed it as a mechanism to maintain a suitable anthocyanin level. The transcriptomic data confirmed that *FLSs* were modulated in berries from water-stressed vines (Savoi et al., 2016), particularly *FLS8*, which was overexpressed in all the deficit irrigated treatments. The *MYBF1*, which has been described as the light-induced transcription factor mainly involved in *FLS* regulation (Czemmel et al., 2009), was particularly upregulated in RDI-1S berries during ripening, but it was not in RDI-LS and RDI-2S. The glycosyltransferases *GT5* and *GT6* catalyzing the glycosylation of quercetins and the rhamnosyltransferase *RhaT1* were all upregulated between irrigation treatments. Despite the lack of analytical data, the transcriptomic analysis suggested a complex modulation of the phenylpropanoid and flavonoid pathways beyond the anthocyanins and flavonols. The RDI treatments generally upregulated important genes such as the *PALs* and *C4Hs* upstream of the pathway. Interestingly, many stilbene synthases were strongly overexpressed by water deficit as confirmed by the related TFs, particularly *MYB14, MYB15, WRKY03*, and *WRKY24*. Less clear were the branches of the pathway related to the hydroxycinnamic acids and flavan-3-ols.

## Conclusions

In conclusion, deficit irrigation applied before veraison induced a high accumulation of anthocyanins, upregulating the expression of many genes of the relative pathway and keeping them overexpressed even when the water deficit was released. Both *F3′H* and *F3′5′H* hydroxylases were overexpressed in RDI-1 berries, however, a higher concentration of di-substituted anthocyanins was observed at harvest. Albeit to a lesser extent, post-veraison water deficit increased the final concentration of anthocyanins, particularly the tri-substituted forms, overexpressing the *F3′5′H* hydroxylases. The water deficit imposed during the lag-phase slightly reduced the anthocyanin content at harvest, showing many downregulations of genes throughout the flavonoid pathway. Either pre- or post-veraison water stress induced a higher anthocyanin accumulation when it was moderate rather than severe, probably due to a higher leaf area and/or photosynthetic activity and to the consequent acceleration of berry ripening processes observed in moderately stressed vines. We also observed that under deficit irrigation conditions, the effect induced by water stress was the main driver to modulate anthocyanin biosynthesis, overwhelming cluster temperature and sunlight exposure which were indirectly modified. Flavonol concentration was higher in pre-veraison severely stressed berries, showing an overexpression of *FLSs* and *GTs*. The higher cluster light exposure induced by water deficit appeared as the key factor to determine flavonol accumulation, even if elevated cluster temperature represented a further limiting factor to their biosynthesis. Thus, our results highlighted that timing and intensities of deficit irrigation can modulate the berry anthocyanin and flavonol content, differently regulating the flavonoid pathway.

## Data availability statement

The original contributions presented in the study are publicly available. This data can be found here: NCBI, PRJNA886074.

## Author contributions

GP participated in the design of the study; carried out the field data acquisition, the laboratory analyses, and part of the transcriptome data analysis; interpreted the results; and developed the manuscript draft and the final version. GC participated in the design of the study, coordinated the field experiments, carried out the field data acquisition, and developed the manuscript draft and the final version. RG participated in the design of the study and critically reviewed the manuscript. CD’O participated in the design of the study, coordinated the laboratory analyses, supervised and elaborated part of the transcriptome data analysis, and developed the manuscript draft and the final version. All authors contributed to the article and approved the submitted version.

## Funding

This research was supported by academic funds from the University of Pisa, Fondazione Bertarelli, and ColleMassari s.p.a.

## Acknowledgments

The authors thank Rolando Calabrò, Massimo Frassi, and Marcello Di Giacomo for their technical assistance.

## Conflict of interest

The authors declare that the research was conducted in the absence of any commercial or financial relationships that could be construed as a potential conflict of interest.

## Publisher’s note

All claims expressed in this article are solely those of the authors and do not necessarily represent those of their affiliated organizations, or those of the publisher, the editors and the reviewers. Any product that may be evaluated in this article, or claim that may be made by its manufacturer, is not guaranteed or endorsed by the publisher.
