# Supplementary material for: Berry flavonoids are differently modulated by timing and intensities of water deficit in Vitis vinifera L. cv. Sangiovese
Source: Front Plant Sci. 2022 Oct 31;13:1040899. doi: 10.3389/fpls.2022.1040899 (PMC9659973; doi:10.3389/fpls.2022.1040899)
Supplement: Supplementary file 1 [file DataSheet_1.docx]

**SUPPLEMENTARY MATERIALS**

**
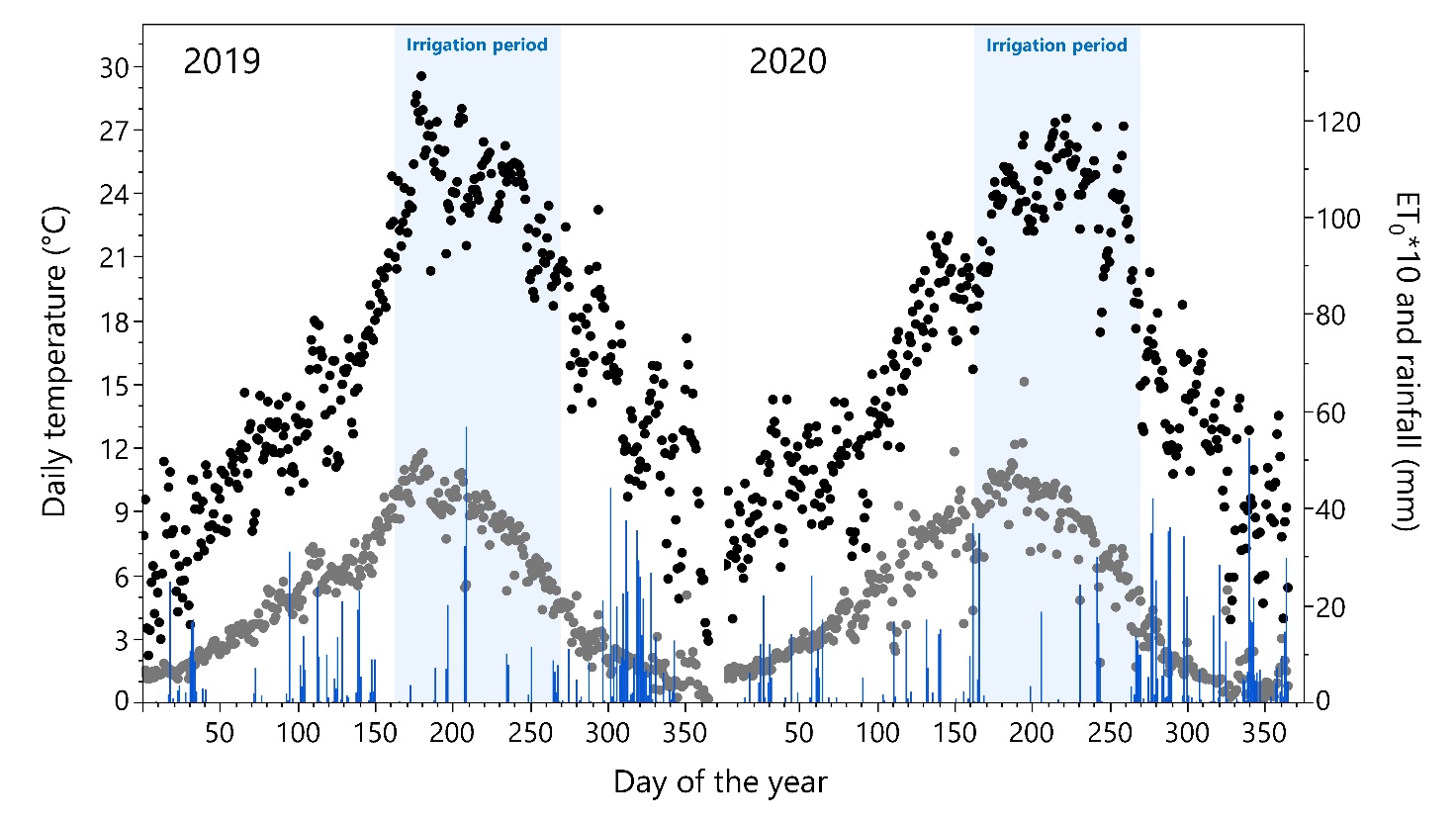
**

**Figure S1.** Daily values of mean air temperature (°C, black dots), evapotranspiration (ET_0_ mm x 10, grey dots) and precipitation (mm, histograms) at the experimental site in 2019 and 2020.

| **Table S2**. Anthocyanins composition measured at different DOY in 2019 and 2020 in berries of Sangiovese grapevines (*Vitis vinifera* L.) subjected to six irrigation regimes. Values (µg g^-1^ of skin fresh weight) are means of three replicates. Different letters indicate significant differences between irrigation treatments after analysis of variance (ANOVA) and Tukey’s HSD test within each year. P values are indicated: *, P < 0.05; **, P < 0.01; ***, P < 0.001; ns (not significant). Legend: GS, glucoside; Ace, acetyl; Caf, caffeoyl; Cou, coumaroyl. | | | | | | | | | | | | | | | | |
| --- | --- | --- | --- | --- | --- | --- | --- | --- | --- | --- | --- | --- | --- | --- | --- | --- |
| DOY  2019/2020 | Compound | 2019 | | | | | |  |  | 2020 | | | | | |  |
|  |  | FI | RDI-1S | RDI-1M | RDI-LS | RDI-2S | RDI-2M | *I* |  | FI | RDI-1S | RDI-1M | RDI-LS | RDI-2S | RDI-2M | *I* |
|  |  |  |  |  |  |  |  |  |  |  |  |  |  |  |  |  |
| 210/207 | Delphinidin-GS | 59.6 cd | 100.4 bc | 206.7 a | 121.7 b | 43.7 d | 43.7 d | *** |  | 81.5 b |  | 160.3 a | 43.3 b | 78.3 b | 83.0 b | *** |
|  | Cyanidin-GS | 171.7 b | 254.5 b | 635.5 a | 182.9 b | 128.0 b | 159.4 b | *** |  | 194.1 b |  | 319.8 a | 104.7 b | 226.8 ab | 188.4 b | ** |
|  | Petunidin-GS | 52.7 cd | 108.9 bc | 203.5 a | 134.8 b | 37.7 d | 38.4 d | *** |  | 71.3 b |  | 162.6 a | 59.0 b | 71.5 b | 93.5 b | ** |
|  | Peonidin-GS | 71.1 cd | 148.4 b | 338.4 a | 116.1 bc | 51.7 d | 67.1 d | *** |  | 58.2 b |  | 127.8 a | 82.0 ab | 89.5 ab | 87.7 ab | * |
|  | Malvidin-GS | 80.9 c | 239.1 b | 382.9 a | 281.6 ab | 59.4 c | 62.1 c | *** |  | 82.7 |  | 113.9 | 121.2 | 112.3 | 64.6 | ns |
|  | Peonidin-Ace-GS | 0.1 b | 0.2 b | 0.5 a | 0.2 b | 0.1 b | 0.1 b | *** |  | 0.2 |  | 0.3 | 0.3 | 0.2 | 0.3 |  |
|  | Malvidin-Ace-GS | 0.2 b | 0.5 ab | 1.0 a | 0.4 b | 0.1 b | 0 b | ** |  | 0.6 |  | 0.5 | 0.4 | 0.6 | 0 |  |
|  | Peonidin-Caf-GS | 0 b | 0.2 ab | 0.4 a | 0.2 ab | 0 b | 0 b | * |  | 0 |  | 0.6 | 0 | 0 | 0 |  |
|  | Malvidin-Caf-GS | 0 | 0 | 0.1 | 0 | 0 | 0 | ns |  | 0 |  | 0.4 | 0 | 0 | 0 |  |
|  | Cyanidin-Cou-GS | 1.0 b | 1.3 b | 4.8 a | 1.0 b | 0.8 b | 0.9 b | ** |  | 0.7 b |  | 1.3 a | 0.6 b | 0.7 b | 0.7 b | *** |
|  | Petunidin-Cou-GS | 0 b | 1.2 a | 0.4 ab | 1.0 a | 0.2 ab | 0 b | ** |  | 0.4 |  | 0.9 | 0.4 | 0.4 | 0.5 |  |
|  | Peonidin-Cou-GS | 0.2 b | 0.5 b | 1.1 a | 0.6 ab | 0 ab | 0 b | ** |  | 0.2 b |  | 0.5 a | 0.4 a | 0.2 b | 0.2 b | ** |
|  | Malvidin-Cou-GS | 0.3 bc | 1.4 abc | 2.4 a | 1.8 ab | 0 bc | 0.4 c | ** |  | 0.7 b |  | 1.5 a | 1.7 a | 0.7 b | 0.6 b | ** |
|  | Total anthocyanins | 438.4 bc | 857.0 b | 1778.4 a | 843.0 b | 322.2 c | 372.5 c | *** |  | 491.0 bc |  | 891.2 a | 414.4 c | 581.6 b | 520.1 bc | * |
|  |  |  |  |  |  |  |  |  |  |  |  |  |  |  |  |  |
| 223/218 | Delphinidin-GS | 314.0 | 296.8 | 382.9 | 347.3 | 363.3 | 329.6 | ns |  | 312.7 ab | 140.1 c | 264.0 ab | 251.0 b | 324.8 a | 327.0 a | *** |
|  | Cyanidin-GS | 655.2 ab | 568.0 ab | 831.8 a | 519.3 b | 727.2 ab | 735.0 ab | * |  | 475.6 bc | 270.3 d | 427.8 bc | 373.9 c | 575.6 a | 579.3 a | *** |
|  | Petunidin-GS | 295.2 b | 300.7 b | 365.0 a | 393.8 a | 350.8 ab | 334.1 ab | ** |  | 289.9 ab | 148.4 c | 267.2 b | 299.5 ab | 333.9 a | 332.1 a | *** |
|  | Peonidin-GS | 332.5 ab | 291.6 b | 402.3 ab | 352.5 ab | 418.0 ab | 452.0 a | * |  | 225.6 cd | 154.9 d | 224.5 cd | 269.3 bc | 369.6 a | 329.5 ab | *** |
|  | Malvidin-GS | 543.9 b | 565.7 b | 658.8 b | 903.9 a | 741.6 ab | 724.9 ab | ** |  | 523.2 bc | 309.7 d | 491.1 c | 754.8 a | 779.5 a | 672.1 ab | *** |
|  | Peonidin-Ace-GS | 0.7 | 0.6 | 0.9 | 0.8 | 0.8 | 0.8 | ns |  | 0.5 | 0.1 | 0.3 | 0.6 | 0.5 | 0.4 | ns |
|  | Malvidin-Ace-GS | 1.6 bc | 1.5 c | 2.1 ab | 2.5 a | 1.9 bc | 1.8 bc | ** |  | 1.4 | 0.4 | 0.7 | 1.9 | 1.8 | 1.8 | ns |
|  | Peonidin-Caf-GS | 0.8 b | 0.7 b | 0.9 b | 1.4 a | 1.0 ab | 1.0 ab | ** |  | 1.2 | 0.6 | 1.2 | 1.9 | 1.0 | 0.9 | ns |
|  | Malvidin-Caf-GS | 0.1 | 0.1 | 0.2 | 0.2 | 0.2 | 0.2 | ns |  | 0.3 | 0.6 | 0.4 | 0.5 | 1.0 | 1.0 | ns |
|  | Cyanidin-Cou-GS | 6.3 ab | 5.4 b | 8.8 a | 8.0 ab | 7.4 ab | 7.4 ab | * |  | 5.5 | 2.1 | 4.1 | 5.2 | 5.4 | 5.4 | ns |
|  | Petunidin-Cou-GS | 1.1 b | 1.0 b | 1.4 b | 1.9 a | 1.3 b | 1.2 b | *** |  | 1.5 b | 0.6 b | 1.5 b | 2.8 a | 1.6 b | 1.6 b | ** |
|  | Peonidin-Cou-GS | 1.9 ab | 1.5 b | 2.6 ab | 3.0 a | 2.7 a | 2.7 a | ** |  | 2.6 | 1.4 | 1.3 | 2.5 | 3.4 | 2.8 | ns |
|  | Malvidin-Cou-GS | 2.3 b | 2.1 b | 2.7 b | 4.5 a | 3.1 b | 2.9 b | *** |  | 1.9 b | 0.8 b | 2.5 ab | 6.8 a | 3.0 ab | 2.8 ab | * |
|  | Total anthocyanins | 2156.3b | 2036.5 b | 2661.1 a | 2539.5 a | 2620.0 a | 2594.3 a | * |  | 1842.6 b | 1030.6 c | 1687.3 bc | 1971.1 b | 2401.9 a | 2257.3 ab | ** |
|  |  |  |  |  |  |  |  |  |  |  |  |  |  |  |  |  |
| 239/230 | Delphinidin-GS | 337.2 b | 383.1 b | 481.3 a | 332.3 b | 410.9 ab | 383.2 b | ** |  | 359.8 b | 430.8 a | 455.8 a | 253.4 c | 336.5 b | 394.5 ab | *** |
|  | Cyanidin-GS | 895.4 b | 972.9 b | 1394.9 a | 907.0 b | 842.9 b | 911.1 b | ** |  | 565.9 b | 500.9 b | 870.0 a | 541.9 b | 612.3 b | 709.5 ab | ** |
|  | Petunidin-GS | 320.8 c | 401.5 ab | 467.4 a | 353.7 bc | 417.9 ab | 409.9 ab | ** |  | 403.8 b | 469.1 ab | 534.2 a | 284.1 c | 397.8 b | 442.9 b | *** |
|  | Peonidin-GS | 479.1 b | 504.1 b | 714.8 a | 476.4 b | 568.0 ab | 626.5 ab | ** |  | 327.8 | 400.8 | 390.7 | 346.9 | 496.9 | 517.2 | ns |
|  | Malvidin-GS | 645.1 b | 854.7 ab | 906.8 ab | 794.3 ab | 1010.5 a | 1017.8 a | * |  | 798.3 bc | 1038.8 ab | 1064.0 a | 711.2 c | 1159.4 a | 1152.2 a | ** |
|  | Peonidin-Ace-GS | 1.4 | 0.7 | 1.0 | 0.8 | 0.7 | 0.9 | ns |  | 0.2 | 0.4 | 0.3 | 0.4 | 0.5 | 0.4 | ns |
|  | Malvidin-Ace-GS | 1.8 | 2.3 | 3.2 | 2.0 | 3.2 | 3.0 | ns |  | 0.9 | 0.5 | 0.9 | 0.3 | 1.2 | 0.6 | ns |
|  | Peonidin-Caf-GS | 0.8 | 1.3 | 1.6 | 1.9 | 2.0 | 1.7 | ns |  | 2.6 | 2.3 | 2.8 | 2.1 | 2.3 | 2.4 | ns |
|  | Malvidin-Caf-GS | 2.1 | 0.8 | 0.8 | 1.3 | 0.4 | 0.4 | ns |  | 1.7 | 2.1 | 2.1 | 1.8 | 1.3 | 1.9 | ns |
|  | Cyanidin-Cou-GS | 10.3 | 9.5 | 14.8 | 13.2 | 9.7 | 10.0 | ns |  | 5.7 | 4.9 | 6.3 | 5.6 | 4.3 | 5.3 | ns |
|  | Petunidin-Cou-GS | 1.6 | 1.9 | 2.2 | 2.0 | 2.1 | 1.9 | ns |  | 2.1 | 1.7 | 1.9 | 2.2 | 1.3 | 2.6 | ns |
|  | Peonidin-Cou-GS | 3.7 | 3.1 | 5.7 | 5.1 | 4.7 | 4.7 | ns |  | 2.2 | 1.8 | 2.1 | 1.9 | 2.3 | 2.5 | ns |
|  | Malvidin-Cou-GS | 3.9 b | 4.3 ab | 5.0 ab | 5.3 ab | 6.9 a | 6.5 ab | * |  | 7.2 | 7.6 | 7.7 | 7.4 | 9.7 | 9.3 | ns |
|  | Total anthocyanins | 2704.0 c | 3140.8 bc | 4000.2 a | 2895.9 bc | 3280.6 b | 3378.2 b | ** |  | 2479.0 bc | 2862.3 b | 3347.3 a | 2159.8 c | 3026.4 ab | 3241.8 a | ** |
|  |  |  |  |  |  |  |  |  |  |  |  |  |  |  |  |  |
| 252/243 | Delphinidin-GS | 351.3 b | 472.3 a | 500.1 a | 334.9 b | 445.8 a |  | ** |  | 358.0 bc | 492.8 a | 491.3 a | 250.9 d | 297.4 cd | 393.7 b | *** |
|  | Cyanidin-GS | 1011.5 c | 1288.9 b | 1631.0 a | 1104.5 bc | 1058.9 bc |  | *** |  | 830.0 cd | 1085.0 ab | 1174.5 a | 859.3 cd | 757.5 d | 968.1 bc | *** |
|  | Petunidin-GS | 345.8 c | 474.7 b | 546.3 a | 351.7 c | 471.4 b |  | *** |  | 371.6 c | 515.0 a | 544.5 a | 275.2 d | 358.2 c | 444.6 b | *** |
|  | Peonidin-GS | 556.1 bc | 706.0 ab | 814.1 a | 493.7 c | 732.5 ab |  | ** |  | 380.8 b | 567.3 a | 591.9 a | 363.8 b | 567.7 a | 642.7 a | *** |
|  | Malvidin-GS | 732.2 c | 1152.3 ab | 957.1 bc | 781.3 c | 1216.0 a |  | *** |  | 735.3 b | 1073.0 a | 1038.7 a | 640.8 b | 1077.2 a | 1133.5 a | *** |
|  | Peonidin-Ace-GS | 1.7 b | 2.3 ab | 2.5 a | 1.6 b | 1.6 b |  | ** |  | 0.9 bc | 1.3 a | 1.3 a | 1.2 ab | 0.7 c | 0.8 bc | ** |
|  | Malvidin-Ace-GS | 2.1 | 1.6 | 1.9 | 1.4 | 2.1 |  | ns |  | 2.0 | 2.5 | 1.9 | 2.0 | 1.0 | 1.4 | ns |
|  | Peonidin-Caf-GS | 2.5 | 4.8 | 4.0 | 3.9 | 3.6 |  | ns |  | 2.1 | 2.4 | 3.7 | 2.3 | 2.6 | 3.2 | ns |
|  | Malvidin-Caf-GS | 1.1 | 3.0 | 2.2 | 2.0 | 2.2 |  | ns |  | 1.8 b | 2.7 a | 2.5 ab | 1.9 b | 2.3 ab | 2.4 ab | ** |
|  | Cyanidin-Cou-GS | 16.6 | 19.1 | 23.2 | 17.3 | 13.9 |  | ns |  | 12.9 bc | 16.3 ab | 18.3 a | 16.1 ab | 10.6 c | 14.0 abc | ** |
|  | Petunidin-Cou-GS | 2.0 | 2.8 | 2.6 | 2.4 | 2.6 |  | ns |  | 2.7 | 3.4 | 3.4 | 3.0 | 2.5 | 3.1 | ns |
|  | Peonidin-Cou-GS | 6.6 | 7.7 | 9.1 | 6.3 | 7.7 |  | ns |  | 4.6 | 5.9 | 6.6 | 5.5 | 5.9 | 7.2 | ns |
|  | Malvidin-Cou-GS | 5.3 | 7.5 | 8.2 | 6.2 | 7.3 |  | ns |  | 6.5 | 7.8 | 7.8 | 7.7 | 8.8 | 9.4 | ns |
|  | Total anthocyanins | 3035.2 c | 4143.5 b | 4502.8 a | 3108.0 c | 3966.2 b |  | ** |  | 2709.8 c | 3775.8 a | 3887.0 a | 2430.5 c | 3093.2 b | 3624.8 a | ** |

| **Table S3**. Flavonols composition measured at different DOY in 2019 and 2020 in berries of Sangiovese grapevines (*Vitis vinifera* L.) subjected to six irrigation regimes. Values (µg g^-1^ of skin fresh weight) are means of three replicates. Different letters indicate significant differences between irrigation treatments after analysis of variance (ANOVA) and Tukey’s HSD test within each year. P values are indicated: *, P < 0.05; **, P < 0.01; ***, P < 0.001; ns (not significant). Legend: GS, glucoside; GN, glucuronide; GA, galactoside; RT, rutinoside. | | | | | | | | | | | | | | | | |
| --- | --- | --- | --- | --- | --- | --- | --- | --- | --- | --- | --- | --- | --- | --- | --- | --- |
| DOY  2019/2020 | Compound | 2019 | | | | | |  |  | 2020 | | | | | |  |
|  |  | FI | RDI-1S | RDI-1M | RDI-LS | RDI-2S | RDI-2M | *I* |  | FI | RDI-1S | RDI-1M | RDI-LS | RDI-2S | RDI-2M | *I* |
|  |  |  |  |  |  |  |  |  |  |  |  |  |  |  |  |  |
| 210/207 | Myricetin-GN | 28.5 b | 44.2 b | 80.7 a | 70.6 a | 24.1 b | 27.9 b | *** |  | 32.2 b |  | 46.9 a | 22.4 c | 26.1 bc | 33.8 b | *** |
|  | Myricetin-GS | 8.3 bc | 19.1 b | 47.2 a | 16.9 bc | 6.6 c | 9.2 bc | *** |  | 9.5 b |  | 15.1 a | 9.5 b | 9.8 b | 11.2 b | ** |
|  | Quercetin-GN | 8.9 c | 30.8 b | 62.3 a | 38.8 b | 7.2 c | 8.4 c | *** |  | 15.9 b |  | 35.4 a | 21.0 b | 15.6 b | 17.5 b | *** |
|  | Quercetin-GA | 356.0 | 382.3 | 360.4 | 399.8 | 371.6 | 376.7 | ns |  | 224.5 |  | 229.4 | 186.7 | 212.4 | 219.8 | ns |
|  | Quercetin-GS | 141.0 ab | 163.4 ab | 331.8 a | 174.1 ab | 145.2 ab | 129.1 b | * |  | 168.8 |  | 152.3 | 82.8 | 124.0 | 131.2 | ns |
|  | Quercetin-RT | 3.9 | 1.9 | 6.6 | 3.4 | 2.8 | 3.0 | ns |  | 0.6 |  | 4.7 | 0.6 | 3.5 | 4.5 | ns |
|  | Kaempferol-GA | 3.2 ab | 5.8 ab | 7.6 a | 4.0 ab | 2.9 ab | 1.3 b | * |  | 3.8 |  | 5.0 | 1.9 | 2.7 | 3.3 | ns |
|  | Kaempferol-GN | 6.4 | 5.5 | 7.0 | 6.3 | 6.1 | 4.3 | ns |  | 2.7 |  | 3.0 | 1.4 | 2.3 | 2.5 | ns |
|  | Kaempferol-GS | 14.3 | 18.5 | 24.8 | 16.4 | 11.3 | 10.1 | ns |  | 11.6 b |  | 29.3 a | 9.4 b | 15.0 ab | 19.2 ab | * |
|  | Isorhamnetin-GA | 4.3 | 6.1 | 6.4 | 5.3 | 2.7 | 4.8 | ns |  | 2.9 |  | 2.9 | 3.6 | 3.1 | 2.8 | ns |
|  | Isorhamnetin-GN | 1.8 | 2.9 | 6.7 | 4.8 | 0.4 | 1.3 | ns |  | 5.4 |  | 0.8 | 0.9 | 1.2 | 0.4 | ns |
|  | Isorhamnetin-GS | 0.3 | 0.4 | 0.5 | 0.5 | 0.4 | 0.2 | ns |  | 0.9 |  | 0.4 | 1.3 | 0.6 | 1.4 | ns |
|  | Total flavonols | 577.6 b | 681.6 b | 942.5 a | 741.4 ab | 581.8 b | 576.9 b | * |  | 479.2 a |  | 525.7 a | 341.7 b | 416.9 ab | 448.1 ab | * |
|  |  |  |  |  |  |  |  |  |  |  |  |  |  |  |  |  |
| 223/218 | Myricetin-GN | 114.6 b | 110.3 b | 114.9 b | 164.6 a | 114.9 b | 117.4 b | ** |  | 104.3 ab | 59.5 b | 76.8 ab | 131.2 ab | 142.7 a | 101.5 ab | * |
|  | Myricetin-GS | 54.3 | 41.1 | 48.8 | 50.3 | 51.8 | 62.4 | ns |  | 33.6 ab | 23.5 b | 26.2 b | 33.5 ab | 47.7 a | 40.5 ab | ** |
|  | Quercetin-GN | 93.5 | 79.8 | 80.2 | 131.1 | 98.8 | 106.0 | ns |  | 82.7 ab | 49.4 b | 62.8 b | 97.5 ab | 133.3 a | 92.6 ab | * |
|  | Quercetin-GA | 445.5 b | 614.9 a | 531.0 ab | 495.3 b | 317.4 c | 292.5 c | *** |  | 540.2 a | 531.6 a | 449.4 ab | 525.7 a | 326.7 b | 294.3 b | ** |
|  | Quercetin-GS | 522.5 b | 618.6 ab | 583.4 ab | 682.8 a | 371.4 c | 349.5 c | *** |  | 450.6 ab | 408.7 ab | 371.1 ab | 569.4 a | 305.8 b | 270.9 b | * |
|  | Quercetin-RT | 7.3 | 7.0 | 6.6 | 10.3 | 6.0 | 5.2 | ns |  | 6.5 | 6.0 | 4.2 | 9.1 | 5.0 | 2.9 | ns |
|  | Kaempferol-GA | 14.0 bc | 17.6 ab | 17.4 ab | 23.4 a | 7.9 cd | 6.8 d | *** |  | 13.3 | 15.1 | 11.3 | 17.0 | 7.5 | 7.3 | ns |
|  | Kaempferol-GN | 13.5 b | 18.3 a | 15.5 ab | 161 ab | 8.4 c | 6.8 c | *** |  | 14.0 | 12.9 | 8.8 | 10.6 | 8.2 | 5.9 | ns |
|  | Kaempferol-GS | 57.4 bc | 74.9 ab | 71.5 ab | 93.4 a | 34.3 c | 28.8 c | *** |  | 45.8 | 51.2 | 47.5 | 47.7 | 18.3 | 21.4 | ns |
|  | Isorhamnetin-GA | 4.2 | 4.0 | 3.7 | 5.1 | 4.3 | 4.0 | ns |  | 3.1 | 2.3 | 2.9 | 6.7 | 7.0 | 6.7 | ns |
|  | Isorhamnetin-GN | 3.8 | 4.2 | 4.8 | 4.8 | 3.3 | 3.5 | ns |  | 3.1 | 2.5 | 2.6 | 4.3 | 6.4 | 1.8 | ns |
|  | Isorhamnetin-GS | 1.3 | 1.7 | 1.5 | 1.9 | 0.9 | 0.8 | ns |  | 0.5 | 0.6 | 0.3 | 1.7 | 1.6 | 1.4 | ns |
|  | Total flavonols | 1332.5 b | 1593.0 a | 1479.9 ab | 1679.6 a | 1019.9 c | 984.1 c | ** |  | 1298.2 ab | 1163.8 b | 1064.5 b | 1454.9 a | 1010.8 b | 847.7 c | ** |
|  |  |  |  |  |  |  |  |  |  |  |  |  |  |  |  |  |
| 239/230 | Myricetin-GN | 99.9 | 102.4 | 95.9 | 120.2 | 141.5 | 127.6 | ns |  | 169.7 | 163.2 | 123.4 | 145.5 | 157.0 | 146.9 | ns |
|  | Myricetin-GS | 61.6 | 52.3 | 71.7 | 58.1 | 62.9 | 61.0 | ns |  | 70.6 a | 46.2 b | 50.3 ab | 69.2 a | 55.8 ab | 51.6 ab | * |
|  | Quercetin-GN | 101.2 | 114.1 | 144.8 | 118.9 | 147.3 | 144.1 | ns |  | 133.8 | 181.2 | 144.9 | 157.0 | 143.1 | 167.3 | ns |
|  | Quercetin-GA | 363.4 bc | 543.9 a | 443.5 b | 417.3 b | 313.1 c | 299.4 c | *** |  | 331.3 bc | 492.3 a | 381.9 ab | 477.7 a | 246.5 bc | 268.9 c | *** |
|  | Quercetin-GS | 667.5 ab | 824.0 ab | 758.4 ab | 881.0 a | 526.9 ab | 477.2 b | * |  | 436.3 ab | 403.8 ab | 423.3 ab | 586.7 a | 250.9 b | 252.8 b | * |
|  | Quercetin-RT | 08.3 | 9.6 | 8.9 | 12.0 | 9.4 | 6.6 | ns |  | 4.7 | 6.0 | 4.0 | 5.5 | 7.3 | 5.3 | ns |
|  | Kaempferol-GA | 14.9 | 23.5 | 18.0 | 16.6 | 8.5 | 9.5 | ns |  | 14.7 | 14.7 | 13.2 | 15.2 | 7.7 | 8.4 | ns |
|  | Kaempferol-GN | 7.5 b | 15.1 a | 7.0 b | 8.3 b | 3.9 b | 4.4 b | *** |  | 11.5 ab | 15.9 a | 13.1 ab | 16.9 a | 7.8 b | 07.6 b | ** |
|  | Kaempferol-GS | 47.8 | 90.2 | 72.4 | 77.8 | 37.0 | 31.1 | ns |  | 45.5 ab | 60.6 a | 52.9 a | 62.9 a | 29.8 b | 30.9 b | *** |
|  | Isorhamnetin-GA | 1.6 | 5.7 | 5.8 | 3.2 | 4.1 | 5.5 | ns |  | 1.7 | 2.4 | 2.8 | 3.6 | 3.4 | 3.2 | ns |
|  | Isorhamnetin-GN | 6.8 | 6.0 | 6.1 | 10.2 | 8.0 | 5.1 | ns |  | 2.7 | 2.4 | 3.2 | 5.9 | 3.9 | 3.6 | ns |
|  | Isorhamnetin-GS | 3.3 | 1.6 | 1.1 | 2.9 | 1.8 | 0.6 | ns |  | 2.7 | 0.6 | 0.7 | 2.4 | 1.0 | 0.2 | ns |
|  | Total flavonols | 1384.4 ab | 1788.9 a | 1634.2 a | 1727.1 a | 1264.8 b | 1172.7 b | * |  | 1225.7 b | 13.9 ab | 1214.3 b | 1549.1 a | 914.6 c | 947.2 c | ** |
|  |  |  |  |  |  |  |  |  |  |  |  |  |  |  |  |  |
| 252/243 | Myricetin-GN | 78.9 | 133.4 | 123.9 | 136.6 | 160.7 |  | ns |  | 137.3 | 119.5 | 132.9 | 99.1 | 108.0 | 135.0 | ns |
|  | Myricetin-GS | 50.0 b | 71.4 ab | 73.4 ab | 53.7 b | 83.7 a |  | ** |  | 56.2 ab | 61.2 ab | 62.2 ab | 47.6 b | 58.9 ab | 71.6 a | * |
|  | Quercetin-GN | 74.0 c | 136.0 ab | 88.6 bc | 89.0 bc | 161.5 a |  | ** |  | 108.7 bc | 149.4 a | 133.2 ab | 90.5 c | 140.2 ab | 159.5 a | ** |
|  | Quercetin-GA | 251.7 c | 475.9 a | 365.5 b | 361.8 b | 321.6 b |  | *** |  | 354.3 bc | 502.7 a | 425.2 ab | 394.0 b | 248.5 c | 337.9 bc | *** |
|  | Quercetin-GS | 577.9 c | 1075.8 a | 900.8 b | 933.5 b | 914.2 b |  | *** |  | 765.5 c | 1069.1 a | 987.6 a | 955.7 ab | 652.1 c | 805.8 bc | *** |
|  | Quercetin-RT | 14.0 b | 21.6 ab | 23.4 ab | 20.4 ab | 29.0 a |  | ** |  | 9.9 | 8.0 | 11.4 | 11.2 | 12.6 | 14.5 | ns |
|  | Kaempferol-GA | 11.1 c | 24.9 a | 17.3 b | 23.3 a | 19.1 b |  | *** |  | 21.4 b | 34.2 a | 26.9 ab | 25.8 ab | 17.8 b | 19.9 b | ** |
|  | Kaempferol-GN | 5.7 | 11.8 | 8.5 | 9.2 | 6.7 |  | ns |  | 9.8 | 9.6 | 8.9 | 7.8 | 5.0 | 7.2 | ns |
|  | Kaempferol-GS | 45.5 c | 103.0 a | 74.8 b | 96.1 a | 77.0 b |  | *** |  | 85.0 bc | 133.6 a | 105.6 ab | 107.3 ab | 60.6 c | 65.5 bc | ** |
|  | Isorhamnetin-GA | 4.2 | 5.9 | 4.0 | 5.4 | 3.3 |  | ns |  | 4.6 | 5.3 | 5.0 | 5.0 | 3.5 | 5.2 | ns |
|  | Isorhamnetin-GN | 6.6 | 7.9 | 6.3 | 7.2 | 6.1 |  | ns |  | 11.3 | 13.1 | 12.5 | 16.0 | 13.9 | 13.2 | ns |
|  | Isorhamnetin-GS | 1.7 | 2.1 | 1.5 | 1.9 | 0.7 |  | ns |  | 3.7 | 4.6 | 3.8 | 4.6 | 4.8 | 4.3 | ns |
|  | Total flavonols | 1121.9 c | 2070.2 a | 1688.5 b | 1738.6 b | 1784.1 b |  | ** |  | 1568.4 bc | 2110.7 a | 1915.8 ab | 1765.3 b | 1326.4 c | 1640.3 b | ** |
